# Mixed phenotype acute leukemia contains heterogeneous genetic mutations by next-generation sequencing

**DOI:** 10.18632/oncotarget.23878

**Published:** 2018-01-03

**Authors:** Andrés E. Quesada, Zhihong Hu, Mark J. Routbort, Keyur P. Patel, Rajyalakshmi Luthra, Sanam Loghavi, Zhuang Zuo, C. Cameron Yin, Rashmi Kanagal-Shamanna, Sa A. Wang, Jeffrey L. Jorgensen, L. Jeffrey Medeiros, Chi Young Ok

**Affiliations:** ^1^ Department of Hematopathology, The University of Texas MD Anderson Cancer Center, Houston, Texas, USA

**Keywords:** mixed phenotype, leukemia, mutations, sequencing

## Abstract

Mixed phenotype acute leukemia (MPAL) is an uncommon manifestation of acute leukemia. The aim of this study is to further characterize the genetic landscape of *de novo* cases of MPAL that fulfill the 2016 World Health Organization (WHO) classification criteria for this entity. We identified 14 cases examined by next generation sequencing (NGS) using 28 (*n =* 10), 53 (*n =* 3) or 81 (*n =* 1) gene panels: 7 cases with a B-cell/myeloid (B/My) immunophenotype, 6 T-cell/myeloid (T/My) immunophenotype, and 1 B-cell/T-cell (B/T) immunophenotype. A total of 25 distinct mutations were identified in 15 different genes in 9/14 (64%) patients. *FLT3*-ITD was the only recurrent mutation in 2 patients. B/My MPAL cases less commonly harbored mutations compared with T/My MPAL cases (43% vs. 100%, *p* = 0.07). In contrast, B/My MPALs more commonly showed a complex karyotype compared to T/My MPALs (71% vs. 17%, *p* = 0.1). With NGS and karyotype combined, most (93%) MPAL cases had mutations or cytogenetic abnormalities. With a median follow-up of 12.5 months, there were no significant differences in median overall survival (OS) between patients with B/My or T/My MPAL (17.8 and 6.5 months, respectively, *p* = 0.81) or between patients with MPAL with versus without gene mutations (6.5 and 13.3 months, respectively, *p* = 0.86). Our data suggest that the distinguishing cases of MPAL according to immunophenotype has value because the underlying mechanisms of leukemogenesis might differ between B/My and T/My MPAL.

## INTRODUCTION

Our capacity to characterize and classify acute leukemia has evolved greatly over recent decades, in part due to advances in technology applied to the study of these neoplasms. Since the advent of immunohistochemistry and flow cytometry, most patients with acute leukemia can be readily assigned to either myeloid, B- or T-lymphoid lineage [1, 2]. However, unusual cases also have been described with confounding immunophenotypes, expressing markers of more than one lineage. In 1981, McGraw *et al.* reported the first case of mixed phenotype acute leukemia (MPAL), followed by a number of other reports, although designated inconsistently using a number of terms in the literature. Reports of MPAL cases prompted the first classification proposal by Catovsky and colleagues in 1991, and followed by subsequent revisions linked to the advent of newer, more specific markers [3–7]. The description of the clinical characteristics, outcomes and various clinicopathologic correlations of MPAL have increased over the last three decades [8–15]. The latest version by the World Health Organization (WHO) included more stringent diagnostic criteria and further delineation of the heterogeneity of cases of MPAL [16, 17].

Mixed phenotype acute leukemia (MPAL) is currently defined as a leukemia in which the blasts express antigens of more than one lineage to such a degree that it is not possible to assign the leukemia to any single lineage with certainty. These cases can be further subdivided into bilineal and biphenotypic. In bilineal MPAL, two distinct blast populations with different immunophenotypes are present. In contrast, biphenotypic MPAL is characterized by one blast cell population expressing markers of more than one lineage [16, 17]. MPAL cases can express either B or T cell antigens together with myeloid markers (B/My or T/My, respectively). Less frequently, rare neoplasms express B and T cell antigens (B/T) or B, T and myeloid antigens (B/T/My) [18, 19].

Despite the progress described above, relatively little is known about the frequencies and types of genetic mutations in MPAL. Of the studies that have assessed MPAL cases for genetic mutations, few have used NGS methods that can assess a large number genes designed to detect common mutations in acute leukemia, including AML and B and T-ALL.

## RESULTS

We identified 14 patients with MPAL, who were examined by an NGS panel. There were 8 (57%) men and 6 (43%) women with a median age of 61 years (range, 19–89 years). There were similar numbers of patients with a B-cell/myeloid (B/My) immunophenotype (7/14, 50%) and a T-cell/myeloid (T/My) immunophenotype (6/14, 43%). There was one patient with a B-cell/T-cell (B/T) immunophenotype. The immunophenotype for each patient is shown in Supplementary Table 4.

Two patients (cases #5 and #7) with B/My MPAL had *BCR-ABL1* rearrangement and one patient (case #2) with B/My MPAL had *KMT2A* (*MLL*) rearrangement. One patient (case #11) with T/My MPAL had *KMT2A* rearrangement. In this cohort the median white blood cell (WBC) count was 4,600/microliter (range, 1,000– 271,200/microliter); the median hemoglobin (Hb) was 9.5 g/dL (range, 5.5–12.8 g/dL); the median platelet count was 76,000/microliter (range, 18,000–275,000 microliter); the median peripheral blood (PB) blast percentage was 15.5% (range, 0–97%); and the median bone marrow (BM) blast percentage was 78.5% (range, 13–92%). BM blasts were higher in patients with T/My than in patients with B/My MPAL (*p* = 0.04) (Table 1). Otherwise, there were no differences were observed in WBC, Hb, and platelet count, and PB blasts between.

**Table 1:** Clinicopathologic features of 14 patients with mixed phenotype acute leukemia

	Total	B/My†	T/My‡	B/T	*P* value († vs. ‡)
**Gender**					
**Male**	8	4	3	1	1
**Female**	6	3	3	0	
**M:F ratio**	1.3	1.3	1	N/A	
**Age**					
**Median (range)**	61 (19–89)	68 (28–89)	57 (19–76)	45	0.19
**WBC (K/uL)**					
**Median (range)**	4.6 (1–271.2)	4.4 (1.3–239.7)	4.9 (1–271.2)	4.8	0.95
**Hemoglobin (g/dL)**					
**Median (range)**	9.5 (5.5–12.8)	9.8 (8.2–12)	9.1 (5.5–12.8)	9.7	0.70
**MCV (fL)**					
**Median (range)**	92.5 (82–103)	92 (85–103)	95 (82–123)	89	0.62
**Platelets (K/uL)**					
**Median (range)**	76 (18–275)	76 (27–268)	105 (18–275)	20	0.86
**PB Blast %**					
**Median (range)**	15.5 (0–97)	7 (0–53)	16 (0–97)	34	0.60
**BM Blast %**					
**Median (range)**	78.5 (13–92)	52 (13–90)	84 (65–92)	84	0.04

B/My, B-cell and myeloid mixed phenotype acute leukemia; T/My, T-cell and myeloid mixed phenotype acute leukemia; B/T, B-cell and T-cell mixed phenotype acute leukemia; WBC, white blood cell count; MCV, mean corpuscular volume; PB, peripheral blood; BM, bone marrow.

**Figure 1:**
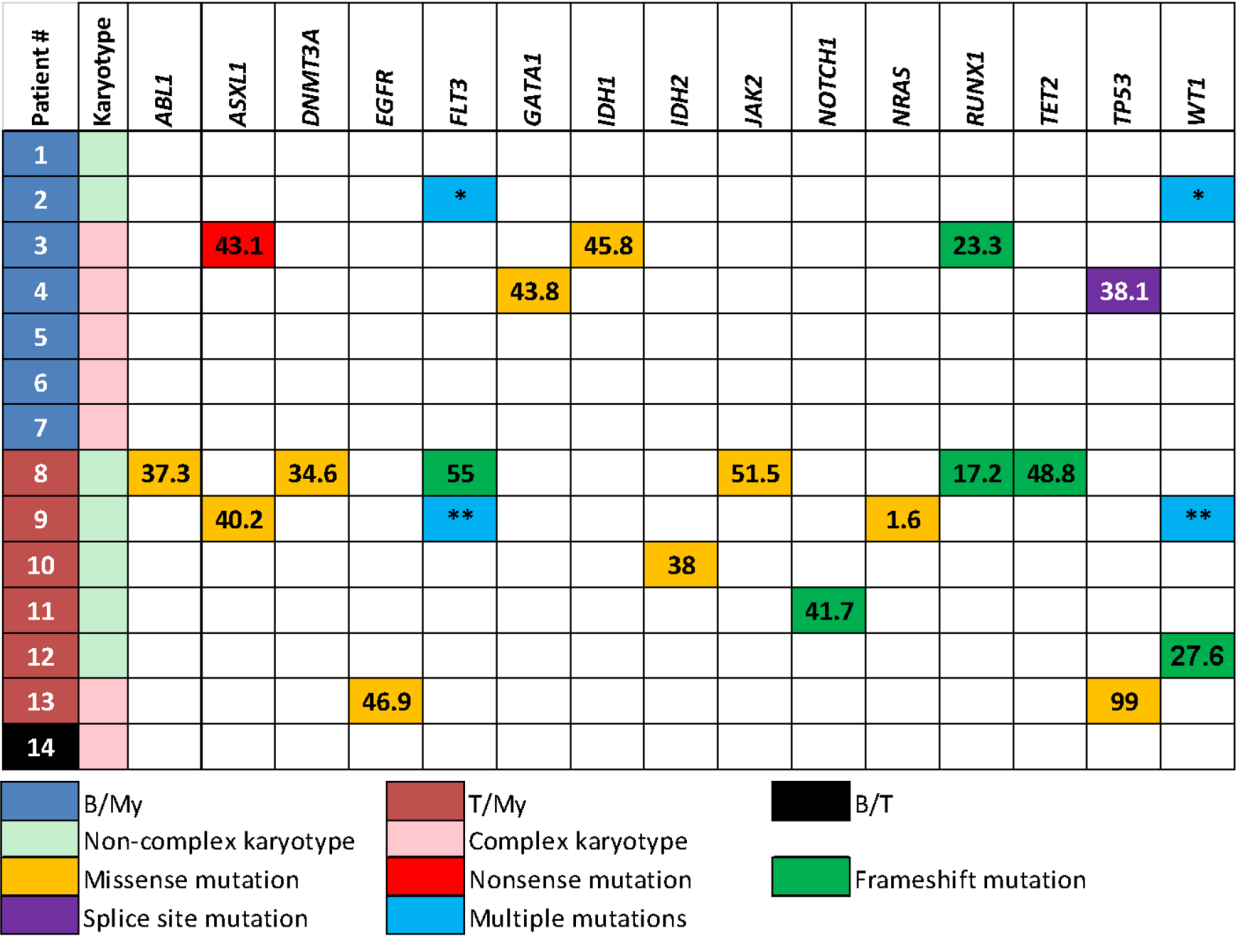
Immunophenotype, karyotype and mutations in each patient with mixed phenotype acute leukemia Patient #2 had two different *in-trans* mutations in *FLT3* and *WT1* genes; *FLT3* p.D835E (mutant allelic frequency, 45%), *FLT3* p.I836fs (4.8%), *WT1* p.R370fs (58.6%) and *WT1* p.V371fs (35.6%). Patient #9 also had multiple mutations in *FLT3* and *WT1* genes; *FLT3*-ITD (29%), *FLT3* p.D835V (11%), *WT1* p.R380fs (8.3%) and *WT1* p.R434fs (38.2%). B/My, mixed phenotype acute leukemia with B-lymphoblast and myeloblast phenotypes; T/My, mixed phenotype acute leukemia with T-lymphoblast and myeloblast phenotypes; B/T, mixed phenotype acute leukemia with B-lymphoblast and T-lymphoblast.^*^: *FLT3* p.D835E (26.4%), *FLT3* p.I836fs (5%), *WT1* p.R370fs (58.6%), *WT1* p.V371fs (35.6%)^**^: *FLT3*-ITD (29%), *FLT3* p.D835V (11%), *WT1* p.R434fs (38.2%), *WT1* p.R380fs (8.3%).

Mutations were detected in 9 (64%) patients; in 5 (36%) patients no mutations were identified. A total of 25 distinct mutations were found involving: *ABL1*, *ASXL1*, *DNMT3A*, *EGFR*, *FLT3*, *GATA1, IDH1*, *IDH2*, *JAK2*, *NOTCH1*, *NRAS*, *RUNX1*, *TET2*, *TP53* and *WT1* (Figure 1). Internal tandem duplications in *FLT3* (*FLT3*-ITD) were the only recurrent mutation (*n* = 2). The median mutant allelic frequency was 38.1% (range, 1.6–99%). When mutations were present they affected at least 2 genes in 6 of 9 patients. Two patients (cases #5 and #7) with t(9;22)/*BCR-ABL1* rearrangement did not have any mutations. Two patients with *KMT2A* rearrangement had mutations (cases #2 and #11). The data suggest that B/My MPAL less commonly harbors mutations than T/My MPAL (43% vs. 100%, *p* = 0.07). Mutant allelic frequencies were similar between the B/My and T/My subtypes (38.1% and 38%, respectively, *p* = 0.76). Two patients (cases #4 and #5) had NGS panels performed subsequently, after therapy. In patient 4, the same *TP53* splice mutation (c.559+1G > A) was detected on 28-gene NGS panel, 3 months apart. The allele frequency seen in the first panel was 38.1% (manual blast count: 52%) and then 11.4% (manual blast count: 13%) in the follow-up panel. No additional mutations were detected at time of subsequent NGS testing. In patient 5, no mutations were detected using a 53-gene NGS panel or using 28-gene panel 8.5 months later.

Conventional cytogenetics showed at least one chromosomal aberration in 10 (71%) patients, including 7 with 3 or more abnormalities (Figure 1 and Table 2). Five of 7 (71%) patients with a B/My immunophenotype had a complex karyotype. In contrast, only 1 (17%) patient with a T/My immunophenotype had a complex karyotype (*p* = 0.1). Thirteen of 14 (93%) patients had either cytogenetic aberrations or gene mutations. Two patients with a B/My immunophenotype also harbored t(9;22)(q34;q11.2)/*BCR-ABL1*, both detected by qualitative multiparametric reverse-transcriptase PCR, quantitative real-time PCR, fluorescence *in situ* hybridization (FISH) and conventional karyotyping. The 2 patients with t(9;22) harbored a complex karyotype. Two patients with a B/My and T/My immunophenotype, respectively, also showed t(v;11q23); *KMT2A* was rearranged detected by FISH and conventional karyotyping.

**Table 2:** Conventional karyotype and treatment in each patient with mixed phenotype acute leukemia

Patient	Age	Sex	NGS	Phenotype	Conventional karyotyping	Treatment
1	28	F	28	B/My	46,XX[20]	CIA, vincristine and dexamethasone
2	38	M	28	B/My	46,XY,t(11;19)(q23;p13.3)[20]	CIA, vincristine and dexamethasone, followed by SCT
3	89	M	28	B/My	49,XY,del(5)(q23q31),del(20)(q11.2q13.3),+21,+21,+21[9]/46,XY[11]	Unknown
4	79	F	28	B/My	46∼55,XX,t(1;7)(q25;q35),del(5)(q13q33),+8,+11,+i(11)(q10), idic(11)(p11.2)x2,add(12)(p12),-15,-16,+19,+r,+3∼4mar[cp17]/46,XX[3]	Fludarabine, cytarabine, vincristine and dexamethasone
5	55	M	53	B/My	46,XY,t(9;22)(q34;q11.2)[9]/47,idem,+der(22)t(9;22)[2]/48,idem,+21,+der(22)t(9;22)[9]	FIA, hyper-CVAD with dasatinib
6	76	F	28	B/My	47,X,del(X)(q22q27),+21[7]/47,XX,+10[2]/46,XX[11]	Hyper-CVAD with inotuzumab
7	85	M	28	B/My	45,X,-Y,inv(1)(p13p36.1)[1]/44,idem,-7,t(9;22)(q34;q11.2)[3]/44,idem,-5,-7, t(9;22),+mar[5]/44,idem,-6,-7,t(9;22),+mar[11]	Hyper-CVAD, dasatinib, rituximab, decitabine
8	76	F	28	T/My	46,XX[20]	Clofarabine and cytarabine
9	57	M	28	T/My	46,XY,t(6;14)(q25;q32)[4]/46,XY[16]	CIA with sorafenib, followed by SCT
10	65	M	53	T/My	46,XY,del(4)(p16)[1]/47,XY,+14[1]/46,XY[18]	Idarubicin, cytarabine, vincristine and dexamethasone, followed by SCT
11	34	F	28	T/My	47,XX,+4,t(11;19)(q23;p13.3)[13]/46,XX[7]	CIA, vincristine and dexamethasone, followed by SCT
12	67	M	81	T/My	46,XY[20]	CIA, vincristine and dexamethasone with plan for future SCT
13	19	F	28	T/My	45,XX,-9,-15,del(16)(p11.2p12.2),+der(?)t(?;9)(?;q32)[10]/45,idem,add(17)(q25)[10]	CIA, vincristine and dexamethasone
14	45	M	53	B/T	50,XY,dup(1)(p22p36.1),+4,+10,-15,+21,+22,+mar[12]/50,idem,del(11)(q12),add(19)(q13.1)[3]/46,XY[5]	Hyper-CVAD, followed by SCT

NGS, next generation sequencing panel used; Hyper-CVAD, cyclophosphamide, vincristine, doxorubicin, dexamethasone, methotrexate and cytarabine; SCT, stem cell transplant; FIA, fludarabine, idarubicin and cytarabine; CIA, clofarabine, idarubicin and cytarabine.

The treatment regimens used for these patients were heterogeneous (Table 2); 8 patients were treated with a hybrid approach (AML-targeted therapy with vincristine and dexamethasone), 3 patients received ALL-targeted therapy (cyclophosphamide, vincristine, doxorubicin, and dexamethasone or its variant), and 2 patients were treated with AML-targeted therapy (clofarabine and cytarabine). The treatment regimen was unknown in one patient. Five patients underwent stem cell transplant by the time of last follow-up.

**Figure 2:**
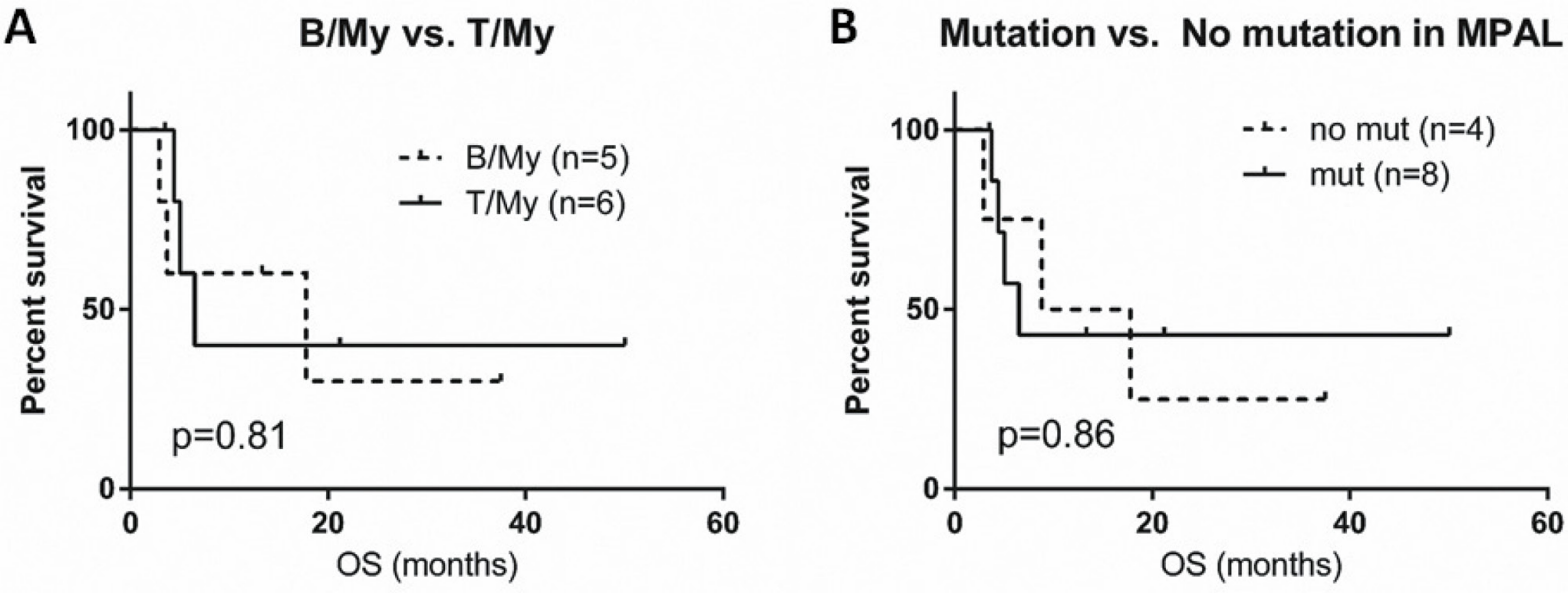
Survival graphs in patients with mixed phenotype acute leukemia (MPAL) (**A**) Overall survival (OS) curve comparison for B/My and T/My MPAL. (**B**) OS curve comparison for MPAL patients with any mutations and patients without detectable mutations. B/My, mixed phenotype acute leukemia with B-lymphoblast and myeloblast phenotypes; T/My, mixed phenotype acute leukemia with T-lymphoblast and myeloblast phenotypes; MPAL, mixed phenotype acute leukemia.

Excluding 2 patients without follow-up information, the median follow-up duration was 12.5 months (range, 2.9 to 54.5 months). The median overall survival (OS) was 7.7 months (range, 2.9 to 50.1 months). Patients with B/My MPAL had longer overall survival (median OS, 17.8 months) compared to patients with T/My MPAL (median OS, 6.5 months), but this difference was not significant (*p* = 0.81) (Figure 2A). A similar trend was seen in patients in whom gene mutations were identified (OS, 6.5 months) compared to patients without any detectable mutations (OS, 13.3 months) but this difference was not significant (*p* = 0.86) (Figure 2B).

## DISCUSSION

The study of ambiguous lineage in cases of acute leukemia continues to be a challenge. The precise cytogenetic and molecular events leading to co-expression of antigens of more than one cell lineage in MPAL are unknown. Cytogenetically, using the 2008 WHO criteria, no single cytogenetic abnormality was overrepresented in a cohort of 100 patients indicating that MPAL is not the result of unique recurrent genetic abnormalities [10]. The outcome of patients with MPAL is inferior to the outcomes of patients with AML or either B or T ALL. Clearly, improved or novel therapies are needed for patients with MPAL. Toward this end, limited preliminary exploration of genetic mutations has been published in patients with MPALs [11, 20–23].

In this study, we found that almost two thirds of cases of MPAL harbor mutations. While our NGS panels cover most genes frequently mutated in AML or ALL (see supplementary information), the true mutation frequency is likely higher because the NGS panels used in this study do not cover genes in cohesion-complex or spliceosome-complex. Accordingly, it is not surprising that a whole exome sequencing study detected mutations in 91% of MPAL cases assessed [23]. Similar to earlier literature, the mutation pattern in patients with MPAL is heterogeneous [11, 23]. Mutated genes involve a variety of cellular functions including chromatin modification (*ASXL1*), DNA methylation (*DNMT3A*, *IDH* and *TET2*), tumor suppressors (*TP53* and *WT1*), transcription factors (*NOTCH1*, *RUNX1*, and *GATA1*) and activated signaling (*FLT3*, *EGFR*, *NRAS* and *JAK2*).

When a gene mutation is present in MPAL, co-mutations of other genes are common (71%). Mutant allelic frequencies in co-mutated genes demonstrate variance, consistent with the heterogeneous nature (i.e. immunophenotype) of MPAL (Figure 1). However, we cannot conclude that MPAL shows a hybrid mutational profile between AML and ALL because many mutations, even those regarded as myeloid-associated gene mutations, are present in both AML and ALL. Particularly, early precursor T-ALL is known to have mutations in *FLT3*, *RAS*, *DNMT3A* and *IDH1/2* [24, 25]. Similarly, *RUNX1* mutations are seen in B-ALL [26]. Interestingly, mutations in *NPM1* and *CEBPA*, which define unique genetic subtypes of AML, were not in the MPAL cases in this study. Absence of mutations in *NPM1* and *CEBPA* also has been observed in other studies [11, 23].

About 70% of MPAL patients have chromosomal aberrations and often have a complex karyotype. Thirteen of 14 (93%) patients had cytogenetic aberrations and/or gene mutations, demonstrating genetic instability in MPAL. There appear to be some differences between B/My and T/My MPAL. B/My MPAL is less commonly associated with gene mutations than T/My MPAL (43% vs. 100%, *p* = 0.07). In contrast, B/My MPAL cases more commonly had a complex karyotype (although not statistically significant). Although the statistical power of this study is insufficient, the data suggest that B/My and T/My cases have disparate pathogenetic mechanisms.

The results in this study and those in the literature show that genomic aberrations in MPAL are complex. Indeed, the few studies reported thus far contain differing results when it comes to genetic mutations seen in MPAL. It is not surprising that there exists such heterogeneity in MPAL considering that the definition of the entity is that of ambiguous lineage. In addition, the pathogenesis of MPAL is not well understood. It is possible that competing transcription factors antagonize the functions of each other to promote expression of one lineage over the other. Alternatively, dysregulation and aberrant expression of transcription factors that govern cell differentiation occur on the basis of the genomic and epigenetic alterations. The timing and level of expression of specific transcription factors may therefore affect lineage determination [27, 28]. For example, reduced expression of human PAX5 during the development of early lymphoid progenitors committed to B lineage has been associated with biphenotypic cells and acute leukemia [29, 30]. It seems that a high level of PAX5 expression is critical for the development of common lymphoid progenitors along the B-cell pathway, whereas low levels may result in a mixed phenotype. Along similar lines, it seems that the fate of early T-cell lineage progenitors is dependent on the Notch receptor signaling pathway, without which myeloid differentiation may occur [31]. This suggestion is consistent with the study by Eckstein *et al.* as well as this study which identified *NOTCH* mutations to only be present in T/My MPAL [23].

The prognosis of patients with MPAL is poor. Although patients were not uniformly treated in this study, we evaluated if mutational status could help to identify patients with a worse outcome. Although not significant, patients with B/My MPAL had a longer median overall survival than patients with T/My MPAL (17.8 and 6.5 months, respectively, *p* = 0.81) (Figure 2A). MPAL patients with mutations also appeared to have a worse OS compared to MPAL patients without mutations (6.5 vs. 13.3 months, *p* = 0.86) (Figure 2B). A larger-scale cohort study is needed to better assess these possible associations.

## MATERIALS AND METHODS

### Diagnostic criteria and patients

We searched for all MPAL cases tested using an NGS panel over 4 years (2012–2016). Acute undifferentiated or unclassifiable leukemia and early thymocyte precursor T-ALL were not included. Molecular data were collected. Clinical, laboratory, cytogenetic and bone marrow findings were also reviewed.

All diagnoses of MPAL were based on the 2008 and 2017 WHO classification criteria. In brief, lineage was designated as follows: 1) myeloid lineage: positive myeloperoxidase (flow cytometry, immunohistochemistry or cytochemistry) or monocytic differentiation (at least 2 of the following: NSE, CD11c, CD14, CD64, lysozyme); 2) T-lineage: positive cytoplasmic CD3 (flow cytometry with antibodies to CD3 epsilon chain) or surface CD3; 3) B-lineage: strong positive CD19 with strong expression of either CD79a, cytoplasmic CD22 or, CD10. Alternatively, a weak CD19 with strong expression of at least 2 of the following: CD79a, cytoplasmic CD22, or CD10.

MPALs were grouped in the following subtypes: 1) B/myeloid, not otherwise specified (NOS), meeting diagnostic criteria for assignment to both B and myeloid lineage, in which the blasts lack genetic abnormalities involving *BCR-ABL1* or *KMT2A*; 2) T/myeloid, NOS, meeting diagnostic criteria for assignment to both T and myeloid lineage, in which the blasts lack genetic abnormalities involving *BCR-ABL1* or *KMT2A* 3) MPAL with t(9,22)(q34;q11.2); *BCR-ABL1* meeting diagnostic criteria for MPAL in which the blasts also have the (9,22) translocation or the *BCR-ABL1* rearrangement; 4) MPAL with t(v;11q23); *KMT2A* rearranged meeting diagnostic criteria for mixed phenotype acute leukemia in which the blasts also have a translocation involving the *KMT2A* gene; and 5) MPAL meeting diagnostic criteria for assignment to both B- and T- lineage. Due to the limited number of cases of MPAL with t(9;22) and MPAL with t(v;11q) we did not analyze these separately, but grouped them by immunophenotype.

### Next generation sequencing

Clinically validated 28-gene, 53-gene or 81-gene panels were used to assess mutational status in all patients as described previously (see supplementary Table 1, 2 and 3 for assessed genes/codons in each panel) [32]. Adequate coverage was defined as ≥ 250 reads for each exon. The analytical sensitivity of the platforms is variable for different genes but is generally 1–3%. Only mutations identified in both forward and reverse reads were considered positive.

### *FLT3* and *CEBPA* analyses

PCR-based DNA analysis was performed to detect internal tandem duplications (ITD) and codon 835/836 point mutations in *FLT3*. A multiplex PCR using fluorescently-labeled primers was performed, followed by detection and sizing of PCR products using capillary electrophoresis. For detecting point mutations in codons 835/836, restriction enzyme digestion of the PCR products was performed prior to capillary electrophoresis. The lower limit of detection (analytical sensitivity) of this assay is approximately 1% of mutant DNA in a background of wild type DNA.

PCR-based DNA sequencing analysis was performed to assess *CEBPA* (CCAAT/enhancer binding protein-alpha). Mutations in this gene have been implicated as a favorable prognostic factor in AML. The lower limit of detection (analytical sensitivity) of the assay is 10–20% mutation bearing cells in the sample tested.

### *BAR-ABL1* analysis

Quantitative real-time PCR analysis was performed on reverse-transcribed RNA for the *BCR-ABL1* fusion transcripts. This multiplex assay is designed to detect common *BCR-ABL1* fusion transcripts e13a2(b2a2), e14a2(b3a2) and e1a2. The fusion transcripts are differentiated based on the size of the PCR product using capillary electrophoresis. *BCR-ABL1* and *ABL1* transcript levels are detected simultaneously and quantitative results are expressed as the percent ratio of *BCR-ABL1* to *ABL1* transcript levels.

### Flow cytometric immunophenotyping

Flow cytometric immunophenotyping was performed in all patients as described previously. [33] The markers assessed included: CD1a, CD2, CD3, CD4, CD5, CD7, CD8, CD10, CD13, CD14, CD15, CD19, CD20, CD22, CD25, CD33, CD34, CD36, CD38, CD41, CD45, CD52, CD56, CD58, CD64, CD79a, CD81, CD123, human leukocyte antigen (HLA)-DR, terminal deoxynucleotidyl transferase (TdT), myeloperoxidase (MPO), cytoplasmic CD3 and cytoplasmic immunoglobulin M (IgM).

### Cytogenetic analysis

Conventional chromosomal analysis was performed on G-banded metaphase cells prepared from unstimulated 24- and 48-hr BM aspirate cultures using standard techniques described previously [34]. Twenty metaphases were analyzed. The karyotype was documented according to the International System for Human Cytogenetic Nomenclature [35].

Fluorescence *in situ* hybridization for detection of *BCR-ABL1* was performed using a LSI fusion probe from Abbott Molecular, Inc (Abbott Park, IL). A total of 200 interphases are analyzed.

Fluorescence *in situ* hybridization for detection of *KMT2A* rearrangement was performed using a LSI dual color, break-apart probe from Abbott Molecular, Inc (Abbott Park, IL) which hybridizes to band 11q23 (spectrum green on the centromeric side and spectrum orange on the telomeric side of the gene breakpoint).

### Statistical analysis

Comparison among categorical variables and numerical variables was carried out by using the Fisher exact test and Mann-Whitney test, respectively. Overall survival was calculated from the date of diagnosis to the date of death or the last date of follow-up, whichever occurs earlier. Patients who underwent allogeneic stem cell transplant were censored. Survival probability was determined using the Kaplan–Meier method, with differences compared by the log-rank test. Statistical analysis was performed using GraphPad Prism (GraphPad software, San Diego, CA) with significance set at a *p*-value < 0.05 (two-sided).

## SUPPLEMENTARY MATERIALS TABLES











## Abbreviations

(MPAL): Mixed Phenotype Acute Leukemia
(NGS): Next generation sequencing
(OS): Overall survival
(AL): Acute leukemia
(WHO): World Health Organization
(AML): Acute myeloid leukemia
(ALL): Acute lymphoblastic leukemia
(B/My): B cell and myeloid immunophenotype
(T/My): T cell and myeloid immunophenotype
(B/T): B cell and T cell immunophenotype
(B/T/My): B cell, T cell and myeloid triple immunophenotype
(WBC): White blood cell
(Hb): Hemoglobin
(PB): Peripheral blood
(BM): Bone marrow

## References

[1] Khalidi HS, Chang KL, Medeiros LJ, Brynes RK, Slovak ML, Murata-Collins JL, Arber DA (1999). Acute lymphoblastic leukemia. Survey of immunophenotype, French-American-British classification, frequency of myeloid antigen expression, and karyotypic abnormalities in 210 pediatric and adult cases. Am J Clin Pathol.

[2] Khalidi HS, Medeiros LJ, Chang KL, Brynes RK, Slovak ML, Arber DA (1998). The immunophenotype of adult acute myeloid leukemia: high frequency of lymphoid antigen expression and comparison of immunophenotype, French-American-British classification, and karyotypic abnormalities. Am J Clin Pathol.

[3] McGraw TP, Folds JD, Bollum FJ, Stass SA (1981). Terminal deoxynucleotidyl transferase-positive acute myeloblastic leukemia. Am J Hematol.

[4] Catovsky D, Matutes E, Buccheri V, Shetty V, Hanslip J, Yoshida N, Morilla R (1991). A classification of acute leukaemia for the 1990s. Ann Hematol.

[5] Bene MC (2009). Biphenotypic, bilineal, ambiguous or mixed lineage: strange leukemias!. Haematologica.

[6] Bene MC, Castoldi G, Knapp W, Ludwig WD, Matutes E, Orfao A, van’t Veer MB (1995). Proposals for the immunological classification of acute leukemias. European Group for the Immunological Characterization of Leukemias (EGIL). Leukemia.

[7] Scamurra DO, Davey FR, Nelson DA, Kurec AS, Goldberg J (1983). Acute leukemia presenting with myeloid and lymphoid cell markers. Ann Clin Lab Sci.

[8] Mirro J, Zipf TF, Pui CH, Kitchingman G, Williams D, Melvin S, Murphy SB, Stass S (1985). Acute mixed lineage leukemia: clinicopathologic correlations and prognostic significance. Blood.

[9] Weir EG, Ali Ansari-Lari M, Batista DA, Griffin CA, Fuller S, Smith BD, Borowitz MJ (2007). Acute bilineal leukemia: a rare disease with poor outcome. Leukemia.

[10] Matutes E, Pickl WF, Van’t Veer M, Morilla R, Swansbury J, Strobl H, Attarbaschi A, Hopfinger G, Ashley S, Bene MC, Porwit A, Orfao A, Lemez P (2011). Mixed-phenotype acute leukemia: clinical and laboratory features and outcome in 100 patients defined according to the WHO 2008 classification. Blood.

[11] Yan L, Ping N, Zhu M, Sun A, Xue Y, Ruan C, Drexler HG, Macleod RA, Wu D, Chen S (2012). Clinical, immunophenotypic, cytogenetic, and molecular genetic features in 117 adult patients with mixed-phenotype acute leukemia defined by WHO-2008 classification. Haematologica.

[12] Legrand O, Perrot JY, Simonin G, Baudard M, Cadiou M, Blanc C, Ramond S, Viguie F, Marie JP, Zittoun R (1998). Adult biphenotypic acute leukaemia: an entity with poor prognosis which is related to unfavourable cytogenetics and P-glycoprotein over-expression. Br J Haematol.

[13] Xu XQ, Wang JM, Lu SQ, Chen L, Yang JM, Zhang WP, Song XM, Hou J, Ni X, Qiu HY (2009). Clinical and biological characteristics of adult biphenotypic acute leukemia in comparison with that of acute myeloid leukemia and acute lymphoblastic leukemia: a case series of a Chinese population. Haematologica.

[14] Al-Seraihy AS, Owaidah TM, Ayas M, El-Solh H, Al-Mahr M, Al-Ahmari A, Belgaumi AF (2009). Clinical characteristics and outcome of children with biphenotypic acute leukemia. Haematologica.

[15] Killick S, Matutes E, Powles RL, Hamblin M, Swansbury J, Treleaven JG, Zomas A, Atra A, Catovsky D (1999). Outcome of biphenotypic acute leukemia. Haematologica.

[16] Borowitz M, Bene MC, Harris NL, Porwit A, Matutes E, Swerdlow SH, Campo E, Harris NL, Jaffe ES, Pileri SA, Stein H, Thiele J, Vardiman JW (2008). Acute leukemias of ambiguous lineage. World Health Organization Classification of Tumours. Pathology and Genetics of Tumours of Haematopoietic and Lymphoid Tissues.

[17] Arber DA, Orazi A, Hasserjian R, Thiele J, Borowitz MJ, Le Beau MM, Bloomfield CD, Cazzola M, Vardiman JW (2016). The 2016 revision to the World Health Organization classification of myeloid neoplasms and acute leukemia. Blood.

[18] Kohla SA, Sabbagh AA, Omri HE, Ibrahim FA, Otazu IB, Alhajri H, Yassin MA (2015). Mixed Phenotype Acute Leukemia with Two Immunophenotypically Distinct B and T Blasts Populations, Double Ph (+) Chromosome and Complex Karyotype: Report of an Unusual Case. Clin Med Insights Blood Disord.

[19] Porwit A, Bene MC (2015). Acute leukemias of ambiguous origin. Am J Clin Pathol.

[20] Heesch S, Neumann M, Schwartz S, Bartram I, Schlee C, Burmeister T, Hanel M, Ganser A, Heuser M, Wendtner CM, Berdel WE, Gokbuget N, Hoelzer D (2013). Acute leukemias of ambiguous lineage in adults: molecular and clinical characterization. Ann Hematol.

[21] Rubnitz JE, Onciu M, Pounds S, Shurtleff S, Cao X, Raimondi SC, Behm FG, Campana D, Razzouk BI, Ribeiro RC, Downing JR, Pui CH (2009). Acute mixed lineage leukemia in children: the experience of St Jude Children’s Research Hospital. Blood.

[22] Wouters BJ, Jorda MA, Keeshan K, Louwers I, Erpelinck-Verschueren CA, Tielemans D, Langerak AW, He Y, Yashiro-Ohtani Y, Zhang P, Hetherington CJ, Verhaak RG, Valk PJ (2007). Distinct gene expression profiles of acute myeloid/T-lymphoid leukemia with silenced CEBPA and mutations in NOTCH1. Blood.

[23] Eckstein OS, Wang L, Punia JN, Kornblau SM, Andreeff M, Wheeler DA, Goodell MA, Rau RE (2016). Mixed-phenotype acute leukemia (MPAL) exhibits frequent mutations in DNMT3A and activated signaling genes. Exp Hematol.

[24] Neumann M, Coskun E, Fransecky L, Mochmann LH, Bartram I, Sartangi NF, Heesch S, Gokbuget N, Schwartz S, Brandts C, Schlee C, Haas R, Duhrsen U (2013). FLT3 mutations in early T-cell precursor ALL characterize a stem cell like leukemia and imply the clinical use of tyrosine kinase inhibitors. PLoS One.

[25] Neumann M, Heesch S, Schlee C, Schwartz S, Gokbuget N, Hoelzer D, Konstandin NP, Ksienzyk B, Vosberg S, Graf A, Krebs S, Blum H, Raff T (2013). Whole-exome sequencing in adult ETP-ALL reveals a high rate of DNMT3A mutations. Blood.

[26] Sood R, Kamikubo Y, Liu P (2017). Role of RUNX1 in hematological malignancies. Blood.

[27] Orkin SH (2003). Priming the hematopoietic pump. Immunity.

[28] Ye M, Iwasaki H, Laiosa CV, Stadtfeld M, Xie H, Heck S, Clausen B, Akashi K, Graf T (2003). Hematopoietic stem cells expressing the myeloid lysozyme gene retain long-term, multilineage repopulation potential. Immunity.

[29] Simmons S, Knoll M, Drewell C, Wolf I, Mollenkopf HJ, Bouquet C, Melchers F (2012). Biphenotypic B-lymphoid/myeloid cells expressing low levels of Pax5: potential targets of BAL development. Blood.

[30] Hsu CL, King-Fleischman AG, Lai AY, Matsumoto Y, Weissman IL, Kondo M (2006). Antagonistic effect of CCAAT enhancer-binding protein-alpha and Pax5 in myeloid or lymphoid lineage choice in common lymphoid progenitors. Proc Natl Acad Sci U S A.

[31] De Obaldia ME, Bell JJ, Wang X, Harly C, Yashiro-Ohtani Y, DeLong JH, Zlotoff DA, Sultana DA, Pear WS, Bhandoola A (2013). T cell development requires constraint of the myeloid regulator C/EBP-alpha by the Notch target and transcriptional repressor Hes1. Nat Immunol.

[32] Ok CY, Patel KP, Garcia-Manero G, Routbort MJ, Peng J, Tang G, Goswami M, Young KH, Singh R, Medeiros LJ, Kantarjian HM, Luthra R, Wang SA (2015). TP53 mutation characteristics in therapy-related myelodysplastic syndromes and acute myeloid leukemia is similar to de novo diseases. J Hematol Oncol.

[33] Zheng W, Medeiros LJ, Young KH, Goswami M, Powers L, Kantarjian HH, Thomas DA, Cortes JE, Wang SA (2014). CD30 expression in acute lymphoblastic leukemia as assessed by flow cytometry analysis. Leuk Lymphoma.

[34] Tang G, Zhang L, Fu B, Hu J, Lu X, Hu S, Patel A, Goswami M, Khoury JD, Garcia-Manero G, Medeiros LJ, Wang SA (2014). Cytogenetic risk stratification of 417 patients with chronic myelomonocytic leukemia from a single institution. Am J Hematol.

[35] McGowan J, Simons A, Schmid M

