# Supplementary material for: Mixed phenotype acute leukemia contains heterogeneous genetic mutations by next-generation sequencing
Source: Oncotarget. 2018 Jan 3;9(9):8441–9. doi: 10.18632/oncotarget.23878 (PMC5823573; doi:10.18632/oncotarget.23878)
Supplement: Supplementary file 1 [file oncotarget-09-8441-s001.pdf]

Mixed phenotype acute leukemia contains heterogeneous genetic mutations by next-generation sequencing

SUPPLEMENTARY MATERIALS

Supplementary Table 1: Genes and codons (exons) covered by a 28-gene panel. See Supplementary_Table_1

Supplementary Table 2: Genes and codons (exons) covered by a 53-gene panel. See Supplementary_Table_2

Supplementary Table 3: Genes and codons (exons) covered by a 81-gene panel. See Supplementary_Table_3

Supplementary Table 4: Immunophenotype by flow cytometry in each patient with mixed phenotype acute leukemia. See Supplementary_Table_4
