# Supplementary material for: Mixed phenotype acute leukemia contains heterogeneous genetic mutations by next-generation sequencing
Source: Oncotarget. 2018 Jan 3;9(9):8441–9. doi: 10.18632/oncotarget.23878 (PMC5823573; doi:10.18632/oncotarget.23878)
Supplement: Supplementary file 2 [file oncotarget-09-8441-s002.docx]

Supplement table 1. Genes and codons (exons) covered by a 28-gene panel

| Gene | Exons (codons) tested |
| --- | --- |
| ABL1 (NM_005157) | 1-3 (1-99), 3-4 (92-200), 4-6 (195-317), 6-10 (307-523), 11 (560-576), 11 (567-606), 11 (598-638), 11 (661-700), 11 (691-731), 11 (723-762), 11 (752-790), 11 (781-820), 11 (810-851), 11 (843-882), 11 (873-912), 11 (935-975), 11 (965-1003), 11 (995-1034), 11 (1070-1131) |
| ASXL1 (NM_015338) | 2-4 (20-104), 4-8 (96-253), 8-11 (250-377), 11 (368-407), 11 (398-437), 11-12 (427-589), 12 (581-619), 12 (640-678), 12 (670-709), 12 (701-740), 12 (732-771), 12 (762-801), 12 (792-831), 12 (821-860), 12 (851-889), 12 (880-919), 12 (911-949), 12 (940-979), 12 (970-1009), 12 (999-1038), 12 (1031-1069), 12 (1061-1100), 12 (1091-1129), 12 (1123-1160), 12 (1153-1191), 12 (1182-1221), 12 (1212-1251), 12 (1241-1281), 12 (1271-1311), 12 (1301-1341), 12 (1336-1375), 12 (1369-1542) |
| BRAF (NM_004333) | 2-3 (47-157), 3-17 (147-709), 18 (729-767) |
| DNMT3A (NM_022552) | 3 (25-59), 4-6 (89-196), 7-8 (214-322), 9-12 (339-486), 12 (477-492), 14-16 (519-641), 17-18 (646-720), 19 (725-739), 19 (729-768), 19-20 (759-803), 22-23 (827-913) |
| EGFR (NM_005228) | 2-3 (30-96), 3-6 (89-249), 7-9 (273-353), 9-12 (343-444), 12-28 (438-1104), 28 (1096-1133), 28 (1171-1211) |
| EZH2 (NM_004456) | 2-10 (1-404), 10-11 (395-466), 11-15 (460-610), 15-19 (606-728), 19-20 (719-752) |
| FLT3 (NM_004119) | 2-3 (15-112), 3-8 (103-311), 8 (301-340), 8-14 (331-610), 14-15 (606-642), 15-24 (635-994) |
| GATA1 (NM_002049) | 2 (1-16), 2-3 (13-89), 3 (81-120), 3 (111-151), 3 (142-182), 4 (200-248), 5-6 (265-306), 6 (297-336) |
| GATA2 (NM_032638) | 2 (1-59), 3 (77-140), 3 (181-220), 3 (212-251), 3 (242-280), 3 (273-291), 4-6 (318-439), 6 (434-474), 6 (466-481) |
| HRAS (NM_005343) | 2-4 (18-129) |
| IDH1 (NM_005896) | 3-4 (1-100), 4 (92-131), 4-6 (121-228), 6-10 (223-415) |
| IDH2 (NM_002168) | 2 (39-69), 3-5 (106-220), 5-7 (212-281), 7 (278-317), 7 (308-323), 9-10 (361-417), 10-11 (408-453) |
| IKZF2 (NM_016260) | 2-4 (1-99), 4 (91-129), 4-8 (121-432), 8 (424-463), 8 (454-494), 8 (484-524), 8 (514-527) |
| JAK2 (NM_004972) | 3-7 (11-220), 7-9 (241-364), 9-11 (359-456), 11-17 (451-723), 17-21 (747-949), 21-25 (939-1133) |
| KIT (NM_000222) | 1-3 (1-128), 3 (119-157), 3-5 (152-292), 5 (253-254), 5-6 (292-321), 6-9 (318-460), 9-10 (454-532), 10-12 (523-606), 12-16 (597-765), 17-18 (788-842), 18-21 (834-977) |
| KRAS (NM_004985) | 2-3 (1-87), 3-5 (81-189) |
| MDM2 (NM_002392) | 1-6 (1-134), 7-11 (143-323), 11 (313-352), 11 (342-379), 11 (370-408), 11 (403-498) |
| MLL (NM_005933) | 2-3 (145-182), 3 (178-215), 3 (206-244), 3 (236-274), 3 (265-303), 3 (293-332), 3 (325-362), 3 (353-392), 3 (390-427), 3 (418-456), 3 (476-515), 3 (506-544), 3 (534-573), 3 (564-603), 3 (594-632), 3 (624-662), 3 (653-691), 3 (682-722), 3 (714-753), 3 (743-780), 3 (771-809), 3 (799-837), 3-5 (831-1129), 5-6 (1121-1212), 7-12 (1218-1507), 13-15 (1526-1617), 15-19 (1610-1805), 19-22 (1796-1943), 22 (1940-1979), 23 (1985-2024), 24-26 (2032-2114), 26-27 (2111-2186), 27 (2176-2214), 27 (2204-2244), 27 (2236-2274), 27 (2264-2303), 27 (2295-2334), 27 (2324-2362), 27 (2354-2392), 27 (2382-2422), 27 (2413-2451), 27 (2443-2480), 27 (2471-2511), 27 (2502-2542), 27 (2533-2570), 27 (2561-2600), 27 (2591-2629), 27 (2619-2660), 27 (2651-2690), 27 (2710-2747), 27 (2798-2836), 27 (2827-2864), 27 (2856-2894), 27 (2885-2924), 27 (2914-2953), 27 (2946-2983), 27 (2974-3012), 27 (3003-3041), 27 (3032-3072), 27 (3063-3102), 27 (3092-3131), 27 (3123-3161), 27 (3152-3190), 27-32 (3183-3729), 32 (3720-3758), 32-36 (3755-3891), 36 (3882-3921), 36 (3918-3970) |
| MPL (NM_005373) | 1 (1-17), 1-3 (7-88), 3 (81-122), 4-7 (146-346), 7 (336-375), 7-9 (370-490), 11 (522-551), 12 (559-636) |
| MYD88 (NM_002468) | 1 (10-50), 1-3 (41-184), 3 (174-213), 3-5 (211-310) |
| NOTCH1 (NM_017617) | 1-3 (1-128), 4 (197-237), 5 (248-289), 6 (318-357), 6 (350-367), 8 (419-443), 8 (434-475), 8-9 (466-504), 10 (519-557), 11-12 (632-672), 13-14 (716-785), 16-17 (823-885), 18 (914-981), 20 (1058-1067), 21 (1109-1166), 21 (1157-1170), 23 (1253-1294), 24 (1335-1338), 25 (1373-1413), 25 (1449-1491), 25 (1481-1523), 25 (1513-1529), 26 (1549-1631), 26 (1625-1666), 27 (1701-1723), 30 (1825-1843), 30 (1835-1874), 30 (1865-1880), 31 (1895-1935), 31 (1965-1978), 32 (2012-2028), 34 (2061-2085), 34 (2131-2210), 34 (2251-2293), 34 (2326-2364), 34 (2356-2398), 34 (2420-2460), 34 (2451-2489), 34 (2481-2521), 34 (2511-2549), 34 (2540-2556) |
| NPM1 (NM_002520) | 1-5 (1-131), 5-11 (123-295) |
| NRAS (NM_002524) | 2 (1-37), 3-5 (77-190) |
| PTPN11 (NM_002834) | 2-3 (5-60), 3-8 (54-299), 9-15 (312-594) |
| RUNX1 (NM_001754) | 3 (20-33), 4 (62-101), 5-6 (118-205), 8-9 (269-435), 9 (428-465), 9 (460-481) |
| TET2 (NM_001127208) | 3 (1-22), 3 (13-52), 3 (44-82), 3 (73-112), 3 (103-140), 3 (132-171), 3 (162-200), 3 (191-229), 3 (220-259), 3 (250-288), 3 (279-318), 3 (308-347), 3 (337-376), 3 (369-408), 3 (399-439), 3 (429-468), 3 (459-498), 3 (488-527), 3 (518-557), 3 (549-586), 3 (577-616), 3 (636-674), 3 (665-704), 3 (694-732), 3 (723-762), 3 (752-790), 3-6 (783-1216), 6-10 (1207-1408), 10 (1399-1438), 10-11 (1432-1533), 11 (1523-1563), 11 (1555-1593), 11 (1585-1622), 11 (1612-1651), 11 (1643-1683), 11 (1674-1712), 11 (1703-1740), 11 (1736-2003) |
| TP53 (NM_000546) | 4 (41-80), 4 (72-112), 4-6 (107-214), 6 (210-224), 7-10 (234-367) |
| WT1 (NM_024426) | 1 (25-63), 1 (163-204), 1 (197-216), 2 (240-257), 4-10 (291-518) |
