# Supplementary material for: Mixed phenotype acute leukemia contains heterogeneous genetic mutations by next-generation sequencing
Source: Oncotarget. 2018 Jan 3;9(9):8441–9. doi: 10.18632/oncotarget.23878 (PMC5823573; doi:10.18632/oncotarget.23878)
Supplement: Supplementary file 3 [file oncotarget-09-8441-s003.docx]

Supplementary table 2. Genes and codons (exons) covered by a 53-gene panel

| Gene | Exons (codons) tested |
| --- | --- |
| ABL1 (NM_007313) | 4-6 (243-362), 7 (395-424) |
| AKT1 (NM_005163) | 3 (16-49) |
| ALK (NM_004304) | 23 (1172-1175), 25 (1248-1275) |
| APC (NM_000038) | 16 (875-918), 16 (1113-1153), 16 (1257-1297), 16 (1288-1328), 16 (1318-1357), 16 (1349-1386), 16 (1377-1575) |
| ATM (NM_000051) | 8 (353-355), 9 (409-412), 12 (601-633), 17 (846-880), 26 (1308-1331), 34 (1678-1719), 35 (1741-1773), 36 (1792-1832), 39 (1940-1973), 50 (2441-2479), 54 (2665-2670), 55 (2694-2717), 56 (2725-2756), 59 (2889-2891), 61 (2946-2950), 63 (3007-3051) |
| BRAF (NM_004333) | 11 (439-471), 15 (581-606) |
| CDH1 (NM_004360) | 3 (77-117), 8 (369-379), 9 (399-439) |
| CDKN2A (NM_000077) | 2 (51-70) |
| CSF1R (NM_005211) | 7 (297-301), 22 (926-970) |
| CTNNB1 (NM_001904) | 3 (12-50) |
| DNMT3A (NM_022552) | 23 (866-913) |
| EGFR (NM_005228) | 3 (108-142), 7 (288-297), 15 (598-627), 18-20 (708-817), 21 (857-875) |
| ERBB2 (NM_004448) | 19 (754-769), 20 (772-818), 21 (839-883) |
| ERBB4 (NM_005235) | 3 (98-140), 4 (153-186), 6 (208-244), 7 (248-287), 8 (295-306), 9 (333-350), 15 (579-619), 23 (907-936) |
| EZH2 (NM_004456) | 16 (618-649) |
| FBXW7 (NM_033632) | 5 (243-278), 8 (375-394), 9 (429-471), 10 (473-508), 11 (549-583) |
| FGFR1 (NM_015850) | 4 (120-126), 7 (247-250) |
| FGFR2 (NM_000141) | 7 (250-311), 7 (302-313), 9 (362-382), 12 (521-550) |
| FGFR3 (NM_000142) | 7 (247-288), 9 (379-422), 14-15 (639-659), 18 (792-807) |
| FLT3 (NM_004119) | 11 (437-456), 14 (569-605), 16 (648-683), 20 (807-843) |
| GNA11 (NM_002067) | 4-5 (172-216), 6-7 (255-360) |
| GNAQ (NM_002072) | 4-5 (159-245), 5-6 (241-297), 6-7 (291-360), 7 (355-360) |
| GNAS (NM_000516) | 8 (200-220) |
| HNF1A (NM_000545) | 3 (205-238), 4 (271-314) |
| HRAS (NM_005343) | 2 (1-15), 3 (38-63) |
| IDH1 (NM_005896) | 4 (90-132) |
| IDH2 (NM_002168) | 4 (125-178) |
| JAK2 (NM_004972) | 14 (615-622) |
| JAK3 (NM_000215) | 13 (568-573), 16 (683-723) |
| KDR (NM_002253) | 6 (220-248), 7 (267-276), 11 (471-476), 19 (872-874), 21 (946-985), 26 (1135-1146), 27 (1171-1211), 30 (1308-1357) |
| KIT (NM_000222) | 2 (51-93), 9-10 (502-547), 10-11 (540-592), 13 (641-664), 14 (670-712), 15 (714-745), 17 (815-828), 18 (838-866) |
| KLHL6 (NM_130446) | 1 (1-98) |
| KRAS (NM_004985) | 2 (1-22), 3 (38-63), 4 (103-147) |
| MET (NM_001127500) | 2 (168-209), 2 (375-400), 14 (1008-1028), 16 (1110-1132), 19 (1247-1284) |
| MLH1 (NM_000249) | 12 (383-426) |
| MPL (NM_005373) | 10 (514-522) |
| NOTCH1 (NM_017617) | 26 (1562-1601), 27 (1673-1679) |
| NPM1 (NM_002520) | 11 (283-295) |
| NRAS (NM_002524) | 2 (1-18), 3 (38-62) |
| PDGFRA (NM_006206) | 12 (552-592), 14 (659-668), 15 (673-717), 18 (823-854) |
| PIK3CA (NM_006218) | 2 (83-118), 5 (345-353), 8 (418-445), 10 (538-555), 14 (701-729), 21 (988-1069) |
| PTEN (NM_000314) | 1 (5-27), 3 (67-70), 6 (170-210), 7 (212-266), 8 (287-342) |
| PTPN11 (NM_002834) | 3 (59-104), 13 (501-533) |
| RB1 (NM_000321) | 4 (127-158), 6 (199-203), 11 (357-376), 17 (550-565), 18 (570-605), 20 (659-700), 21 (703-733), 22 (746-775) |
| RET (NM_020975) | 10-11 (610-667), 13 (766-798), 15 (880-910), 16 (918-934) |
| SMAD4 (NM_005359) | 3 (119-142), 5 (167-208), 6 (243-263), 8 (310-319), 9 (329-373), 10 (385-424), 11 (443-480), 12 (496-535) |
| SMARCB1 (NM_003073) | 2 (39-78), 4 (156-167), 5 (199-210), 9 (381-386) |
| SMO (NM_005631) | 3 (197-242), 5 (323-366), 6 (403-422), 9 (533-551), 11 (639-646) |
| SRC (NM_005417) | 14 (530-537) |
| STK11 (NM_000455) | 1 (36-77), 4-5 (193-211), 6 (261-288), 8 (332-370) |
| TP53 (NM_000546) | 2 (1-12), 4 (69-112), 5 (126-186), 5-6 (181-192), 6 (187-223), 6-7 (214-253), 8 (267-306), 10 (332-342) |
| VHL (NM_000551) | 1 (88-114), 2 (129-155), 3 (157-200) |
| XPO1 (NM_003400) | 14-15 (501-575) |
