# Supplementary material for: Mixed phenotype acute leukemia contains heterogeneous genetic mutations by next-generation sequencing
Source: Oncotarget. 2018 Jan 3;9(9):8441–9. doi: 10.18632/oncotarget.23878 (PMC5823573; doi:10.18632/oncotarget.23878)
Supplement: Supplementary file 4 [file oncotarget-09-8441-s004.docx]

Supplementary table 3. Genes and codons (exons) covered by a 81-gene panel

| Gene | Exons (codons) tested |
| --- | --- |
| ANKRD26 (NM_014915) | 1 (1-6) |
| ASXL1 (NM_015338) | 11-12 (362-1442), 12 (1450-1542) |
| ASXL2 (NM_018263) | 11-12 (381-1436) |
| BCOR (NM_017745) | 2-4 (1-511), 4-6 (515-1080), 7 (1090-1098), 7-12 (1122-1547), 13-15 (1550-1644), 15 (1663-1722) |
| BCORL1 (NM_021946) | 1-6 (1-1261), 6 (1292-1323), 6-8 (1326-1487), 9-11 (1491-1600), 11-12 (1606-1700), 12 (1706-1712) |
| BRAF (NM_004333) | 11 (439-478), 15 (581-620) |
| BRINP3 (NM_199051) | 2-8 (1-767) |
| CALR (NM_004343) | 9 (352-418) |
| CBL (NM_005188) | 7-9 (336-477) |
| CBLB (NM_170662) | 7-10 (282-469) |
| CBLC (NM_012116) | 7-9 (336-454), 10 (465-475) |
| CEBPA (NM_004364) | 1 (1-113), 1 (249-358), 1 (215-244), 1 (128-175), 1 (178-201) |
| CREBBP (NM_004380) | 1-8 (1-608), 9-31 (615-1943), 31 (1950-2443) |
| CRLF2 (NM_022148.2) | 6 (217-256) |
| CSF3R (NM_156039) | 14 (575-622), 17 (681-864) |
| CUX1 (NM_181552) | 2-6 (11-172), 6-9 (174-241), 10-14 (248-408) |
| DDX41 (NM_016222) | 1-17 (1-623) |
| DNMT3A (NM_022552) | 8-22 (286-862), 23 (866-913) |
| EED (NM_003797) | 1-2 (1-69), 2-8 (71-287), 9-12 (289-442) |
| ELANE (NM_001972) | 1-2 (1-48), 2 (69-75), 3-5 (93-268) |
| ETNK1 (NM_018638) | 3 (228-275) |
| ETV6 (NM_001987) | 1-8 (1-453) |
| EZH2 (NM_004456) | 2-5 (1-158), 5-6 (160-205), 7 (209-217), 8-19 (243-732), 20 (752) |
| FBXW7 (NM_033632) | 9-12 (413-708) |
| FLT3 (NM_004119) | 11-20 (437-847) |
| GATA1 (NM_002049) | 2-3 (1-84) |
| GATA2 (NM_032638) | 2-5 (1-377), 5-6 (379-481) |
| GFI1 (NM_005263) | 2 (2-39) |
| GNAS (NM_000516) | 8 (200-202), 11 (315-324) |
| HNRNPK (NM_002140) | 3-17 (1-465) |
| HRAS (NM_005343) | 2-3 (1-70), 3-4 (74-150) |
| IDH1 (NM_005896) | 4 (132-133) |
| IDH2 (NM_002168) | 4 (125-178) |
| IKZF1 (NM_006060) | 2-8 (1-443), 8 (445-518) |
| IL2RG (NM_000206) | 1-2 (1-45), 2-8 (51-370) |
| IL7R (NM_002185) | 5-7 (180-292) |
| JAK1 (NM_002227) | 3-22 (3-1023), 22-24 (1026-1123) |
| JAK2 (NM_004972) | 10 (405-442), 12-14 (505-622), 16 (665-711), 18 (762-805) |
| JAK3 (NM_000215) | 2-23 (1-1069) |
| KDM6A (NM_021140) | 1-8 (1-218), 9-19 (228-971), 19-21 (977-1070), 22-29 (1080-1402) |
| KIT (NM_000222) | 8-9 (411-514), 11 (550-592), 17 (788-828) |
| KMT2A (NM_005933) | 2 (145-168), 3-4 (176-1075), 4 (1081-1112), 5 (1117-1184), 6 (1190-1212), 7 (1224-1325), 8-10 (1338-1440), 11-13 (1445-1560), 14-15 (1566-1665), 27 (2186-2195), 27 (2201-2355), 27 (2373-3215), 27 (3223-3324), 27 (3339-3575) |
| KRAS (NM_004985) | 2-4 (1-150) |
| MAP2K1 (NM_002755) | 2 (27-90), 3 (98-146) |
| MPL (NM_005373) | 10 (490-522), 12 (552-636) |
| NF1 (NM_001042492) | 1-5 (1-189), 6 (201-218), 8-13 (244-467), 13-22 (478-992), 23-24 (997-1066), 25-26 (1082-1146), 26-31 (1160-1378), 31-35 (1382-1550), 35-38 (1564-1868), 39 (1870-1884), 39-47 (1886-2322), 47-52 (2325-2555), 52 (2568-2575), 53-58 (2580-2840) |
| NOTCH1 (NM_017617) | 26-28 (1529-1795), 34 (2061-2286), 34 (2290-2556), 34 (2061-2286), 34 (2290-2556) |
| NPM1 (NM_002520) | 11 (283-295) |
| NRAS (NM_002524) | 2-4 (1-150) |
| PAX5 (NM_016734) | 1-10 (8-392) |
| PHF6 (NM_032458) | 2-3 (1-78), 4-10 (81-366) |
| PIGA (NM_002641) | 2 (1-6), 2-3 (16-283), 4-5 (294-396), 6 (399-485) |
| PML (NM_033238) | 3 (201-255) |
| PRPF40B (NM_001031698) | 2-19 (2-609), 19-20 (611-658), 20-26 (661-893) |
| PTEN (NM_000314) | 7-8 (212-280), 8 (310-339) |
| PTPN11 (NM_002834) | 3-4 (46-125), 7 (253-285), 12 (460-462), 12-13 (465-533) |
| RAD21 (NM_006265) | 2-14 (1-632) |
| RARA (NM_000964) | 6-7 (211-338) |
| RUNX1 (NM_001754) | 2-9 (1-438), 9 (456-474) |
| SETBP1 (NM_015559) | 4 (838-885) |
| SF1 (NM_004630) | 1-13 (1-640) |
| SF3A1 (NM_005877) | 1-9 (1-424), 9-16 (427-794) |
| SF3B1 (NM_012433) | 13-16 (574-790) |
| SH2B3 (NM_005475) | 2 (1-121), 2 (129-170), 2 (189-205), 2-8 (210-576) |
| SMC1A (NM_006306) | 1-7 (1-415), 8-24 (419-1206), 25 (1211-1234) |
| SMC3 (NM_005445) | 1 (1-5), 2-6 (19-110), 6-16 (113-504), 16-17 (507-580), 17-25 (591-975), 25-27 (979-1151), 28-29 (1159-1217) |
| SRSF2 (NM_003016) | 1 (1-38), 1 (45-121) |
| STAG1 (NM_005862) | 2 (1-5), 3-12 (10-395), 13-22 (402-738), 22-27 (740-953), 27-34 (955-1259) |
| STAG2 (NM_006603) | 2-15 (1-512), 16-20 (541-699), 21-32 (714-1219), 33 (1225-1232) |
| STAT3 (NM_139276) | 17-22 (489-715) |
| STAT5A (NM_003152) | 3-7 (1-214), 8-9 (249-295), 9-20 (303-795) |
| STAT5B (NM_012448) | 16 (636-693) |
| SUZ12 (NM_015355) | 1-2 (17-107), 4-5 (129-169), 7-16 (198-740) |
| TERC (NR_001566) | non-coding RNA |
| TERT (NM_198253) | 1 (1-24), 1-2 (33-172), 2-4 (246-630), 4-16 (633-1133) |
| TET2 (NM_001127208) | 3 (1-77), 3 (91-826), 3 (829-853), 3-11 (867-2003) |
| TP53 (NM_000546) | 2 (1-25), 4 (33-34), 4-11 (80-394) |
| U2AF1 (NM_006758) | 2 (15-44), 6 (117-161) |
| U2AF2 (NM_007279) | 1-5 (1-161), 6-12 (163-473), 12 (475-476) |
| WT1 (NM_024426) | 1 (122-216), 1 (2-59), 1 (70-93), 2-10 (216-518) |
| ZRSR2 (NM_005089) | 1-3 (1-68), 4 (71-90), 6-8 (134-201), 8 (212-257), 10-11 (276-435), 11 (440-483) |
